# Effect of Panax notoginseng Saponins on Focal Cerebral Ischemia-Reperfusion in Rat Models: A Meta-Analysis

**DOI:** 10.3389/fphar.2020.572304

**Published:** 2021-02-09

**Authors:** Tao Sun, Ping Wang, Ting Deng, Xingbao Tao, Bin Li, Ying Xu

**Affiliations:** ^1^ Department of Pharmacology, College of Pharmacy, Chengdu University of Traditional Chinese Medicine, Chengdu, China; ^2^ College of Pharmacy, Nanjing University of Chinese Medicine, Nanjing, China; ^3^ Hospital of Chengdu University of Traditional Chinese Medicine, Chengdu, China

**Keywords:** *Panax notoginseng* saponins, ischemic stroke, middle cerebral artery occlusion, focal cerebral ischemia-reperfusion, animal model

## Abstract

With the increase of the aging population, the high mortality and disability rates caused by ischemic stroke are some of the major problems facing the world, and they dramatically burden the society. *Panax notoginseng* (Burk) F. H. Chen, a traditional Chinese medicine, is commonly used for promoting blood circulation and removing blood stasis, and its main bioactive components are *Panax notoginseng* saponins (PNS). Therefore, we performed a meta-analysis on focal cerebral ischemia-reperfusion animal models established with middle cerebral artery occlusion (MCAO) surgery to evaluate the therapeutic effect of PNS. We systematically searched the reports of PNS in MCAO animal experiments in seven databases. We assessed the study quality using two literature quality evaluation criteria; evaluated the efficacy of PNS treatment based on the outcomes of the neurological deficit score (NDS), cerebral infarct volume (CIV), and biochemical indicators via a random/fixed-effects model; and performed a subgroup analysis utilizing ischemia duration, drug dosage, intervention time, and administration duration. We also compared the efficacy of PNS with positive control drugs or combination treatment. As a result, we selected 14 eligible studies from the 3,581 searched publications based on the predefined exclusion-inclusion criteria. PNS were significantly associated with reduced NDS, reduced CIV, and inhibited release of the inflammatory factors IL-1β and TNF-α in the focal MCAO rat models. The PNS combination therapy outperformed the PNS alone. In addition, ischemia time, drug dosage, intervention time, and administration duration in the rat models all had significant effects on the efficacy of PNS. Although more high-quality studies are needed to further determine the clinical efficacy and guiding parameters of PNS, our results also confirmed that PNS significantly relieves the focal cerebral ischemia-reperfusion in rat models. In the animal trials, it was suggested that an early intervention had significant efficacy with PNS alone or PNS combination treatment at a dosage lower than 25 mg/kg or 100–150 mg/kg for 4 days or longer. These findings further guide the therapeutic strategy for clinical cerebral ischemic stroke.

## Introduction

As the population ages, stroke, especially ischemic stroke, has become a serious disease that threatens human life (Benjamin et al., 2018). The global prevalence of cerebrovascular disease was 80.1 million people in 2016. Of these patients, 67.6 million were affected by ischemic stroke (Benjamin et al., 2018; Benjamin, 2020). There was a 2.7% increase in the prevalence of ischemic stroke and a 1.7% decrease in the prevalence of hemorrhagic stroke from 2006 to 2016. Two point seven (2.7) million cases died of ischemic stroke and 2.8 million died of hemorrhagic ischemic stroke. Based on comparisons in the past few decades, the prevalence and mortality rates of stroke have generally declined in high-income countries, but no significant changes have been found in low- and middle-income countries (Nogles and Galuska, 2020). This situation is likely related to advancements in the awareness, treatment, and control rates of modifiable risk factors in higher-income countries. In the United States (US), nearly 795,000 people suffer from a new or recurrent stroke each year, with 87% having experienced ischemic strokes, 10% having experienced intracerebral hemorrhage (ICH) strokes, and 3% having experienced subarachnoid hemorrhage (SAH) strokes. In China, stroke is the leading cause of death among all other causes (Li et al., 2019). The incidence of hemorrhagic stroke has decreased by 1.7% annually, and the ischemic stroke has increased by 8.7% from 1984 to 2004 (Boehme et al., 2017).

The middle cerebral artery (MCA) is the most common artery involved in acute ischemic stroke (AIS) (Nogles and Galuska, 2020), which causes severe nerve damage and poor prognosis. Studies (Ng et al., 2007) have shown that the two most severe ischemic strokes are those in more than one vascular territory (MVT) and MCA. These strokes both cause severe neurocognitive impairments encompassing executive functions, memory, attention, and behavioral issues, such as depression, agitation, and abulia (van Lieshout et al., 2019; Busk et al., 2020; Chen et al., 2020; Draaisma et al., 2020; Syafrita et al., 2020). MCA strokes may even lead to further visuospatial cognitive impairments and impaired language. The small proportion of patients with MCA stroke who are discharged home is most probably because of reduced main functional measure scores and increased disability (Ng et al., 2007). Both treatment and rehabilitation for stroke pose enormous economic cost and cause severe nursing and financial burdens to patients and families (Johnson et al., 2019).

Early effective prevention and emergency treatment can maximize the prevention of stroke and minimize stroke cerebral nerve injury. The three broad levels of stroke prevention (Boehme et al., 2017) are primordial prevention to form a healthy lifestyle, primary prevention to protect nonstroke and transient ischemic attack (TIA) individuals from risk factors, and secondary prevention to prevent stroke recurrence. The suitable treatments for an acute ischemic stroke may be medical thrombolysis (intravenous alteplase) within 4.5 h, antiplatelet therapy within 12 h, endovascular thrombectomy (EVT) within 24 h, and early management of patients considered for hemicraniectomy within 48 h (Boulanger et al., 2018; Nogles and Galuska, 2020). There is no curative therapy if the treatment window is missed, and treatments will only improve symptoms and prevent relapse (Nogles and Galuska, 2020).

Although the conventional treatment has significant effects on acute ischemic stroke, ethnic medicine also plays an important role, especially in the field of traditional Chinese medicine (TCM). TCM plays an essential role in the diagnosis, prevention, and treatment of patients suffering from AIS in China for more than 2,000 years. Modern scholars of TCM also point out that blood stasis is the principal problem in the process of occurrence, development, and outcome of ischemic stroke (Wang et al., 2014; Zhong et al., 2015; Sun et al., 2016; Chai and Zhang, 2018). Therefore, in the prevention (Chang et al., 2016; Tsai et al., 2016; Song et al., 2019), treatment (Chen et al., 2019; Hsu et al., 2019; Luo et al., 2019), and rehabilitation (Huang et al., 2018; Zhao et al., 2018) of ischemic stroke, conventional treatment combined with drugs that promote blood circulation and remove blood stasis can be an effective intervention.

In the clinical practice of TCM, Chinese herbal medicine is generally considered nontoxic and potentially therapeutic for patients and is extensively used in stroke patients, although it is lacking in high-quality supportive literature (Yu et al., 2019; Wei et al., 2020). *Panax notoginseng* (Burkill) F. H. Chen (*P. notoginseng*), as well as its extract components or pharmaceutical preparations, is a commonly used medicine for promoting blood circulation and removing blood stasis (He et al., 2011; Gao et al., 2013; Gui et al., 2013; Liu et al., 2014a; Luo et al., 2018). *P. notoginseng* has platelet aggregation inhibition, anti-inflammation, and antioxidation effects and improves the absorption of intracranial hematoma and promotes the recovery of nerve function. Given the high morbidity and mortality of stroke, animal models have been developed over 4 decades to replicate as many aspects of human stroke as possible to further understand the underlying pathophysiological response and to explore the potential treatments (Kwolek-Klimkiewicz et al., 1996; Makkiyah and Sadewo, 2019; Wei et al., 2019).

**FIGURE 1:**
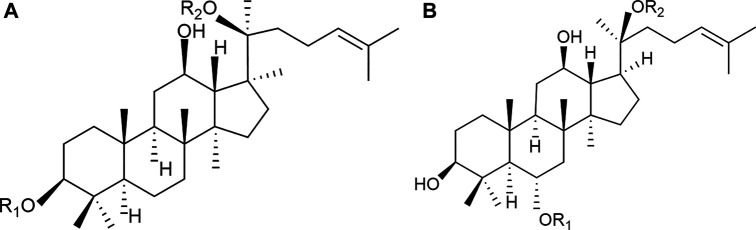
Chemical structures of the main saponins in PNS. The main five saponins were divided into two different dammarane-type saponins, namely, **(A)** 20(S)-protopanaxatriol saponins (PTS), including notoginsenoside R_1_, ginsenoside Rg_1_, and Re, and **(B)** 20(S)-protopanaxadiol saponins (PDS), including ginsenoside Rb_1_ and Rd.

*Panax notoginseng* saponins (PNS) are recognized as the main bioactive ingredients of *P. notoginseng*, including different dammarane-type saponins (Yoshikawa et al., 2003; Xu et al., 2019). A series of commercial medicinal products of PNS-related preparations have been widely used in China, such as Xuesaitong injections, Xuesaitong capsules, Xuesaitong granules, and Xueshuantong capsules. According to the *Chinese Pharmacopeia* (Chinese Pharmacopoeia Commission, (2015)) for PNS-related preparations, the main chemical constituents of PNS contain five dammarane-type saponins, including notoginsenoside R_1_, ginsenoside Rg_1_, ginsenoside Re, ginsenoside Rb_1_, and ginsenoside Rd (Figure 1 and Table 1). The total content of the five ingredients is not less than 75% by high-performance liquid chromatography (HPLC) (Table 1).

**TABLE 1:** The main chemical components isolated from PNS and/or its preparations.

Chemical component	Molecular formula	IUPAC name	Content criteria*
Notoginsenoside R_1_	C_47_H_80_O_18_	(2S,3R,4S,5S,6R)-2-[(2S)-2-[(3S,5R,6S,8R,9R,10R,12R,13R,14R,17S)-6-[(2R,3R,4S,5S,6R)-4,5-dihydroxy-6-(hydroxymethyl)-3-[(2S,3R,4S,5R)-3,4,5-trihydroxyoxan-2-yl]oxyoxan-2-yl]oxy-3,12-dihydroxy-4,4,8,10,14-pentamethyl-2,3,5,6,7,9,11,12,13,15,16,17-dodecahydro-1H-cyclopenta[a]phenanthren-17-yl]-6-methylhept-5-en-2-yl]oxy-6-(hydroxymethyl)oxane-3,4,5-triol	≥5.0%
Ginsenoside Rg_1_	C_42_H_72_O_14_	(2R,3R,4S,5S,6R)-2-[[(3S,5R,6S,8R,9R,10R,12R,13R,14R,17S)-3,12-dihydroxy-4,4,8,10,14-pentamethyl-17-[(2S)-6-methyl-2-[(2S,3R,4S,5S,6R)-3,4,5-trihydroxy-6-(hydroxymethyl)oxan-2-yl]oxyhept-5-en-2-yl]-2,3,5,6,7,9,11,12,13,15,16,17-dodecahydro-1H-cyclopenta[a]phenanthren-6-yl]oxy]-6-(hydroxymethyl)oxane-3,4,5-triol	≥25.0%
Ginsenoside Re	C_48_H_82_O_18_	(2S,3R,4R,5R,6S)-2-[(2R,3R,4S,5S,6R)-2-[[(3S,5R,6S,8R,9R,10R,12R,13R,14R,17S)-3,12-dihydroxy-4,4,8,10,14-pentamethyl-17-[(2S)-6-methyl-2-[(2S,3R,4S,5S,6R)-3,4,5-trihydroxy-6-(hydroxymethyl)oxan-2-yl]oxyhept-5-en-2-yl]-2,3,5,6,7,9,11,12,13,15,16,17-dodecahydro-1H-cyclopenta[a]phenanthren-6-yl]oxy]-4,5-dihydroxy-6-(hydroxymethyl)oxan-3-yl]oxy-6-methyloxane-3,4,5-triol	≥2.5%
Ginsenoside Rb_1_	C_54_H_92_O_23_	(2R,3R,4S,5S,6R)-2-[[(2R,3S,4S,5R,6S)-6-[(2S)-2-[(3S,5R,8R,9R,10R,12R,13R,14R,17S)-3-[(2R,3R,4S,5S,6R)-4,5-dihydroxy-6-(hydroxymethyl)-3-[(2S,3R,4S,5S,6R)-3,4,5-trihydroxy-6-(hydroxymethyl)oxan-2-yl]oxyoxan-2-yl]oxy-12-hydroxy-4,4,8,10,14-pentamethyl-2,3,5,6,7,9,11,12,13,15,16,17-dodecahydro-1H-cyclopenta[a]phenanthren-17-yl]-6-methylhept-5-en-2-yl]oxy-3,4,5-trihydroxyoxan-2-yl]methoxy]-6-(hydroxymethyl)oxane-3,4,5-triol	≥30.0%
Ginsenoside Rd	C_48_H_82_O_19_	(2S,3R,4S,5S,6R)-2-[(2R,3R,4S,5S,6R)-2-[[(3S,5R,6S,8R,9R,10R,12R,13R,14R,17S)-3,12-dihydroxy-4,4,8,10,14-pentamethyl-17-[(2S)-6-methyl-2-[(2S,3R,4S,5S,6R)-3,4,5-trihydroxy-6-(hydroxymethyl)oxan-2-yl]oxyhept-5-en-2-yl]-2,3,5,6,7,9,11,12,13,15,16,17-dodecahydro-1H-cyclopenta[a]phenanthren-6-yl]oxy]-4,5-dihydroxy-6-(hydroxymethyl)oxan-3-yl]oxy-6-(hydroxymethyl)oxane-3,4,5-triol	≥5.0%
Total (Notoginsenoside R_1_+ Ginsenoside Rg_1_+ Ginsenoside Re+ Ginsenoside Rb_1_+ Ginsenoside Rd)			≥75.0%

* The content of saponin components in PNS and its preparations refers to the Chinese Pharmacopeia (Chinese Pharmacopoeia Commission, (2015)).

Based on a large-scale trove of experimental animal data, this study systematically evaluated and meta-analyzed the efficacy of PNS in middle cerebral artery occlusion (MCAO) animal models with focal cerebral ischemia-reperfusion (I/R).

## Methods

### Review Protocol

This article is a meta-analysis based on Reporting Items for Systematic Reviews and Meta-Analyses (PRISMA). We registered the review protocol at the International Prospective Register of Systematic Reviews (PROSPERO) (registration no. CRD42020182383).

### Search Strategy

We selected relevant studies from the publications of February 2020 in PubMed, Embase, Medline, Web of Science, Chinese National Knowledge Infrastructure (CNKI), and Wanfang Data information site. The language was restricted to Chinese and English. The subject terms for the Chinese literature search were *Panax notoginseng* (三七) and ischemia (缺血) OR cerebral ischemia (脑缺血). The MeSH terms and text words of *Panax notoginseng*, brain ischemia, stroke, and cerebrovascular disorder, were used to search English literature combinatorially. After the document retrieval strategy was formulated, two investigators (Tao Sun and Xing-Bao Tao) independently performed document retrieval. Disputes in the selection process were discussed and resolved together if any.

### Inclusion and Exclusion Criteria

This systematic evaluation investigated the pharmacodynamics of PNS in animal models with focal cerebral I/R. According to the literature title, abstract, and full-text article, we rated the studies as eligible based on the predetermined inclusion and exclusion criteria (Table 2). The inclusion and exclusion criteria were based entirely on, but not limited to, animals, interventions, comparators, outcomes, and study designs. In this work, a professional document management software (Endnote X9) was used by two investigators independently, and disagreements were addressed by a third investigator (YX).

**TABLE 2:** Inclusion and exclusion criteria.

Principle	Inclusion criteria	Exclusion criteria
Animals	1. Focal MCAO animal models without restriction on the ischemia duration and reperfusion duration.	1. Animal models with noncerebral ischemia, whole-brain ischemia, and ischemia-nonperfusion.
Interventions	2. The PNS (including extracts containing different parts of *P. notoginseng*) and its preparations have an accurate dosage. 3. The concomitant medication of PNS with certain Chinese medicinal ingredients or therapeutic methods.	2. Intervention drugs of *P. notoginseng*, non-PNS ingredients of *P. notoginseng*, *P. notoginseng* prescription without accurate dose, etc.
Comparators	4. Studies with a control group and data should be extracted if a positive group existed.	3. Experimental design without a control group.
Outcomes	5. Outcomes with NDS measured according to Longa et al. (1989) criteria. Under the premise, the other outcome data should be extracted, including CIV and biochemical indicators.	4. Outcomes without NDS measured according to Longa et al. (1989) criteria, only CIV measurements, physiological or biochemical outcomes of treatment.
Study designs	6. Randomized controlled studies with at least seven experimental animals in each group	5. Each group with seven sample sizes below.
Others	/	6. Case reports, clinical trial studies, abstracts, editorials, reviews, conference abstracts, duplication data, and incomplete text.

### Quality Assessment

The SYRCLE’s Risk of Bias tool was adopted to evaluate the risk of bias for all included study (Hooijmans et al., 2014), including (1) random sequence generation; (2) baseline characteristics; (3) allocation concealment; (4) random housing; (5) blinded investigators; (6) blinded outcome assessment; (7) blinded outcome; (8) incomplete outcome data; (9) selective outcome reporting; and (10) ethical considerations. As the weights of the ten entries might vary depending on the outcome and review, it is challenging to justify the weights assigned. The total score inevitably assigned the “weights” to specific domains in the tool. Therefore, we just evaluated the items rather than summarizing them.

In the present study, we examined the animal models with cerebral ischemia-reperfusion, and we employed a revised tool of the Collaborative Approach to Meta-Analysis and Review of Animal Data from Experimental Studies (CAMARADES) (Macleod et al., 2004) checklist to assess the quality of the included studies. The leading evaluation indicators include (1) peer-reviewed publications; (2) presence of randomization of subjects into treatment groups; (3) assessment of dose-response relationship;(4) blinded assessment of behavioral outcome; (5) monitoring of physiological parameters such as body temperature; (6) calculation of necessary sample size to achieve sufficient power; (7) statement of compliance with animal welfare regulations; (8) avoidance of anesthetic agents with marked intrinsic neuroprotective properties; (9) statement of potential conflict of interests; and (10) use of a suitable animal model.

If the study was exclusively described as “completed as an experimental article” and “carried out taking into concern previous experiments,” the quality evaluation basis was further analyzed. If the reference belonged to the same team, the modeling data in the reference were directly extracted and considered of “low risk”; otherwise, it was considered “unclear risk.” The above two evaluation methods were consistent in determining the same indicators and were assessed by two investigators (PW and TD), and the differences were resolved by discussion.

### Data Extraction

The following data were extracted from the included literature: (1) document elements: first author and year of publication; (2) experimental animal elements: animal species, sex, and weight; (3) MCAO model elements: anesthetic type and dose, number of experimental animals, ischemia duration, and reperfusion duration; (4) intervention elements: intervention group, administration route, dosage, intervention time point, and treatment duration; and (5) experimental outcomes: each outcome indicator.

In this meta-analysis study, neurological deficit score (NDS) and cerebral infarct volume (CIV) were used as the effect-quantity index, whereas the animal biochemical experiment indicators were used as possible drug mechanisms. If several independent intervention groups were included, all were included as study groups (e.g., different doses of PNS intervention groups in the same control group). In the control group, samples were divided into subgroups without affecting the sample size. If the data from the study literature were incomplete or only image data were displayed, the original data were discussed with the author. If necessary, data were estimated from graphics or recalculated from available data. The article was removed due to lack of exact data. The data were extracted from each article by two investigators independently.

### Statistical Analysis

A meta-analysis was performed for NDS reported in 10 or more articles. For subgroup analysis, a minimum of two studies per subgroup was required. The NDS, CIV, and biochemical indicators were analyzed as continuous variables. We calculated the pooled estimates of the mean differences (SD)/standardized mean difference (SMD) between PNS and control groups, together with 95% confidence interval (CI). We used the Cochran *Q* test and I^2^ testing to assess heterogeneity between studies. To further explore the potential efficacy and pharmacological mechanism of PNS, we analyzed the drug effects of the PNS group, PNS combination group, and positive drug group on NDS and/or CIV indicators.

Heterogeneity was considered significant at *p* < 0.1. I^2^ testing values of 25, 50, and 75% were considered of low, moderate, and high inconsistency, respectively. When the heterogeneity was moderate or high, the sensitivity analyses of the literature study were investigated and conducted with subgroup analysis. The preset factors for the subgroup analysis included ischemia duration (1, 2 h, and unknown), drug dosage (D_1_ < 25 mg/kg, 25 mg/kg ≤ D_2_ < 50 mg/kg, 50 mg/kg ≤ D_3_ < 100 mg/kg, and 100 mg/kg ≤ D_4_ < 150 mg/kg), intervention time [(1) before occlusion, representing primordial prevention; (2) before and after occlusion, representing primary prevention; and (3) after occlusion, representing secondary prevention], and administration duration (1–3, 4–7, and >7 days). When the heterogeneity was strong and could not be eliminated, the random-effect model was employed to obtain an overall MD/SMD and 95% CI instead of the fixed-effect model. The funnel plot and Egger’s test were used to detect publication bias. In case of any deviation, the trim-and-fill computation was used to detect potential publication bias. All the analyses were performed with RevMan 5.3 and STATA 14.0.

## Results

### Study Selection

**FIGURE 2:**
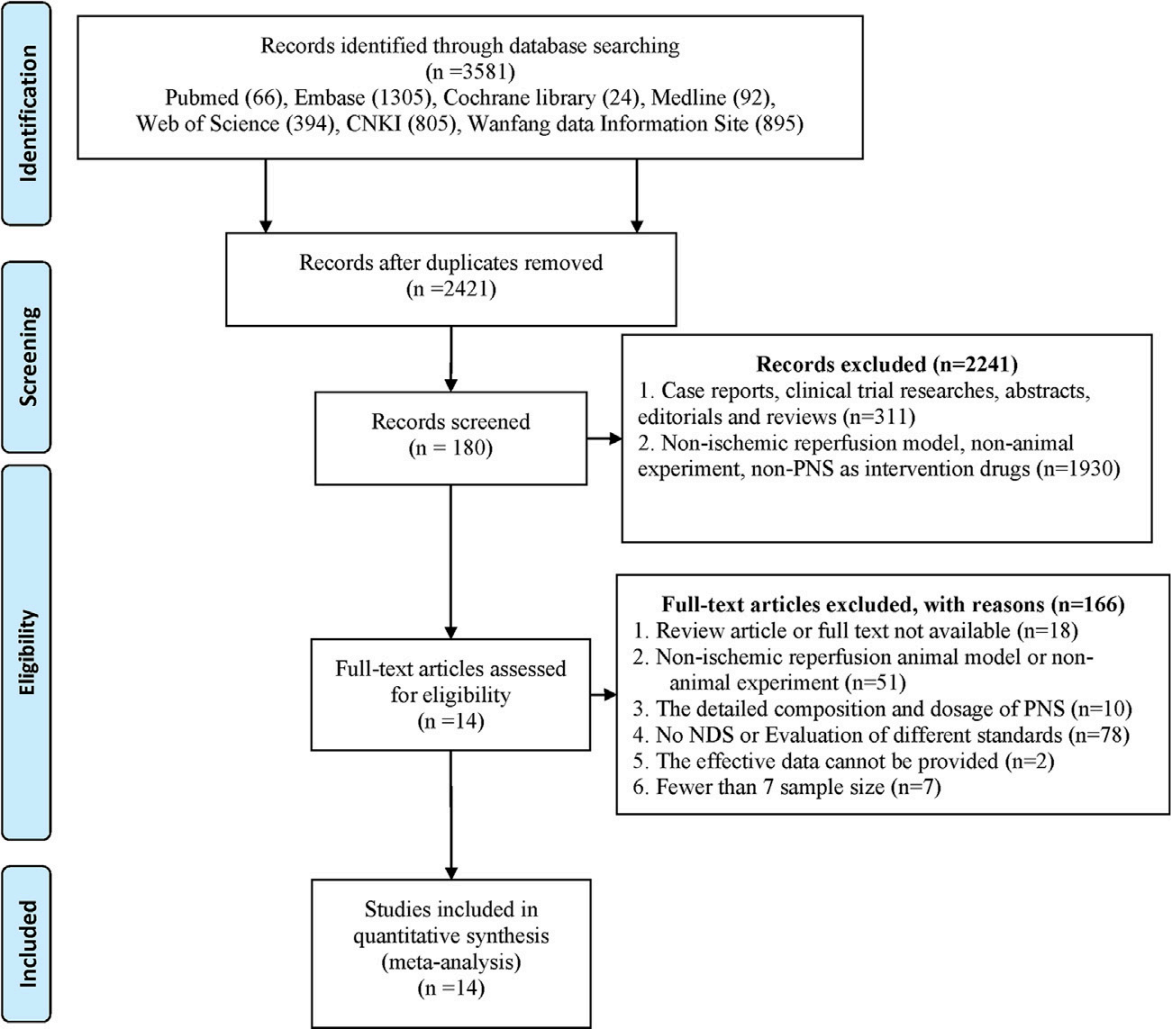
Flow diagram of the Reporting Items for Systematic Reviews and Meta-Analyses (PRISMA). We screened all studies in the database based on the search terms and set the exclusion criteria in advance. The first round of screening was based on the title and abstract of the publication. We carefully studied the full text to make a judgment where insufficient data from the relevant abstracts were available for our decision.

We identified a total of 3,581 search results. Six hundred and fifty-nine (659) duplications were ruled out by software (Endnote X9), and 501 duplications were manually removed. According to the inclusion and exclusion criteria, a total of 2,241 articles were deleted based on full-text reading, title and abstract. Among these articles, 1,930 failed to meet the requirements for experimental subjects, intervention measures, and experimental methods. Through full-text reading and reconfirmation in 180 articles, 166 articles were excluded because they were nonstandard. Among them, 18 were reviewed or not available, 61 subjects or interventions were inconsistent, 78 outcome indicators failed to fulfill the inclusion criteria, 2 had documented valid data that could not be provided, and 7 had small sample size and did not meet the requirements. A total of 14 studies were included in the meta-analysis (Figure 2).

### Study Characteristics

A total of 14 studies were included in this study, and all were published in Chinese (Table 3). Among the well-established studies, all the experimental animal models were rats, and 11/14 (78.57%) used only male rats. Seven of fourteen (7/14) included the experiments in which the rats were anesthetized with chloral hydrate without apparent neuroprotective effects, and the remaining seven articles did not illustrate the type of anesthetic drug used. In the included studies, nine articles (64.29%) had cerebral ischemia for 2 h and two articles (14.29%) for 1 h, and the remaining three articles had no specific ischemic duration. Three publications studied the effects of different reperfusion time on the effective index of the rat model after ischemia (Liu et al., 2014b; Meng et al., 2014; Lin et al., 2019). Of the 14 studies, six compared the medication of positive control drugs (nimodipine, edaravone, and ligustrazine), two compared the efficacy of concomitant medication (*Crataegus pinnatifida* Bge. leaf extract and astragalosides IV), and six compared different dosage groups of PNS (four articles with positive drug comparison and two articles with concomitant medication at dosage of 10–144 mg/kg). Regarding the route of administration, 10 studies included intraperitoneal injections (71.43%), two included intragastric administration, one included intravenous injection (Sun et al., 2010), and one did not clearly state the route of administration (Jiang et al., 2015). In terms of drug intervention time points, one article conducted a study on the time point of intervention (Chen et al., 2007), three articles had administration before occlusion, four articles had administration after occlusion, and six articles had administration before and after occlusion. Additionally, five articles used Xuesaitong injection (lyophilized), one study used *P. notoginseng* flower saponins (PNFS) (Liu et al., 2018), and one study used *P. notoginseng* leaves saponins (PNLS) (He et al., 2017). Eight of the articles used 2,3,5-triphenyltetrazolium chloride (TTC) to measure the CIV, and only five quantitatively analyzed the percentage. Table 4 shows a summary of the preparations used in the 14 selected articles.

**TABLE 3:** Characteristics of the included studies.

Study		Year	Animal species		Model (Ischemia Duration and Anesthetics)		Intervention							
							Group (N, Mass Fraction, Route)	Dosage		Time		Outcome Items/Time/Sample size)	
Chen Jie Chen et al. (2015)		2015	SD rats, 260–300 g, M		MCAO (ZL), 2 h, 10% Chloral hydrate (3.5 ml/kg)		PNS (*n* = 20, ip)	PNS 100 mg/kg		15 min before and 6 h after occlusion		1. NDS (ZL, 22 h) 2. CIV (2% TTC, 22 h) 3. The content of Evans blue 4.IL-1β, TNF-α in brain tissue	
Chen Ming Chen et al. (2007)		2007	SD rats, 250–300 g, M		MCAO (ZL), 2 h, NR		1. PNS-30 min before occlusion (*n* = 8, ip) 2. PNS-2 h after occlusion (*n* = 8, ip) 3. PNS-30 min before and 2 h after occlusion (*n* = 8, ip)	PNS 50 mg/kg PNS 50 mg/kg PNS 50 mg/kg*2		30 min before occlusion 2 h after occlusion 30 min before and 2 h after occlusion		1. NDS (ZL, 0.5/1/4/8/24 h) 2. p53 protein in the ischemic region by immunohistochemistry	
He Yan He et al. (2017)		2017	Wistar rats, 200–220 g, F/M		MCAO (ZL), 1 h, Chloral hydrate (300 mg/kg, ip)		1. PNS (*n* = 8, ig) 2. SS combination-H (*n* = 8, ig) 3. SS combination-M (*n* = 8, ig) 4. SS combination-L (*n* = 8, ig) 5. PNLS (*n* = 8, ig) 6. Hawthorn leaf extract groups (*n* = 8, ig)	PNS 42 mg/kg SS combination-H 24.5 mg/kg SS combination-M 12.25 mg/kg SS combination-L 6.125 mg/kg PNLS 10.5 mg/kg Hawthorne P.E 14 mg/kg		Drug was administered at 7 days before occlusion, and surgery was performed at 5 days after administration		1. NDS (ZL, 48 h) 2. CIV (2% TTC, 24 h) by pathological morphology	
Jiang Huihui Jiang et al. (2015)	2015		SPF SD rats, 180–220 g, M		MCAO (ZL), 2 h, 10% Chloral hydrate (350 mg/kg)	1. PNS (*n* = 12, NR) 2. Nimodipine (*n* = 12, NR)		PNS 25 mg/kg Nimodipine 1 mg/kg		7 days before surgery		1. NDS (ZL, 24 h) 2. CIV (2% TTC, 24 h) by pathological morphology 3. VEGF	
Li Jingxian Li et al. (2017)	2017		SPF SD rats, 220–250 g, M		MCAO (ZL), 2 h, 10% Chloral hydrate (300 mg/kg, ip)	1. AST IV (*n* = 8, above 98%, ig) 2. PNS (*n* = 8, above 98%, ig) 3. AST IV+PNS-H (*n* = 8, ig) 4. AST IV+PNS-L (*n* = 8, ig)		AST IV 28 mg/kg PNS 80 mg/kg AST IV 56 mg/kg+ PNS 160 mg/kg AST IV 28 mg/kg+ PNS 80 mg/kg		administered 2 days before occlusion		1. NDS (ZL, 24 h) 2. CIV (2% TTC, 24 h) by pathological morphology 3. The contents of AST IV, Rg1, Rb1, R1 in rat plasma of different time points	
Lin Jun Lin et al. (2019)	2019		SPF SD rats, 250–300 g, M		MCAO (ZL), 2 h, NR	PNS (subgroup *n* = 12, ip) include 4 subgroups: sacrificed 24/72 h/7 days/3 weeks after reperfusion		PNS 50 mg/kg		4 h after surgery until the sacrifice time		1. NDS (ZL, 24/72 h/7 days/3 weeks) 2. Ultramicrostructure of NVU in the peri-ischemic brain tissue by electron microscopy (24/72 h/7 days/3 weeks) 3. NeuN, GFAP and LN (24/72 h/7 days/3 weeks)	
Liu Lixing Liu et al. (2014b)	2014		SD rats, 280–320 g, M		MCAO (ZL), NR,10% Chloral hydrate (10 ml/kg, ip)	1. PNS-H (subgroup *n* = 8, ip) 2. PNS-L (subgroup *n* = 8, ip) 3. Nimodipine (subgroup *n* = 8, ig)		PNS 7.2 mg/(100 g·d) PNS 3.6 mg/(100 g·d) Nimodipine 1.44 mg/(100 g·d)		5 h after the surgery for 3/7/14/28 days		1. NDS (ZL, 5/24/48/72 h) 2. PSD-95, Syp in brain tissue (3/7/14/28 days)	
Liu Yinhua Liu et al. (2018)	2018		Wistar rats, 280–320 g, M		MCAO (ZL), NR, NR	1. PNFS-H (*n* = 12,72%, ip) 2. PNFS-L (*n* = 12,72%, ip) 3. Nimodipine (subgroup *n* = 8, ig)		PNFS 200 mg/kg*72%=144 mg/kg PNFS 100 mg/kg*72%=72 mg/kg Nimodipine 1.44 mg/(100 g·d)		14 days before occlusion		1. NDS (ZL,24 h) 2. The cerebral histopathology was detected by HE staining 3. The levels of NSE in serum, IL-10, TNF-α, IL-1β 4. The level of Bcl-2, Bax, Caspase-3 in brain homogenate by ELISA	
Meng Lanqing Meng et al. (2014)	2014		SPF Wistar rats, 220–260 g, NR		MCAO (ZL), 2 h, 10% Chloral hydrate (350 mg/kg, ip)	PNS (subgroup n = 10, ip) include two subgroups: be sacrificed 24 h/7 days after reperfusion		PNS 30 mg/kg		At the beginning of reperfusion until the corresponding time point		1. NDS (ZL, 24 h/7 days) 2. The expressions of Laminin, GFAP, and NeuN (24 h/7 days)	
Sun Xiaoqing Sun et al. (2010)	2010		Wistar rats, 270–300 g, F/M		MCAO (ZL), 1 h, NR	1. PNS-L (*n* = 8, iv) 2. PNS-M (*n* = 8, iv) 3. PNS-H (*n* = 8, iv) 4. Nimodipine (subgroup *n* = 8, iv)		PNS 30 mg/kg PNS 60 mg/kg PNS 120 mg/kg Nimodipine 0.4 mg/kg		One half was used for ischemia and one half for reperfusion		1. NDS (ZL, 24 h) 2. CIV (4% TTC, 24 h) (compensation for cerebral swelling in the ischemic hemisphere was considered)	
Tang Jingshu Tang and Pei (2011)	2011		SD rats, 260–300 g, M		MCAO (ZL), 2 h, 10% Chloral hydrate (3.5 ml/kg, ip)	PNS (*n* = 28, ip)		PNS 100 mg/kg		15 min before and 6 h after surgery		1. NDS (ZL, 22 h) 2. CIV (2% TTC, 22 h) 3. BBB 4. IL-1β, TNF-α, IL-6, IL-8 in brain tissue	
Wang Jiehua Wang (2014)	2014		SD rats, 250–280 g, M		MCAO (ZL), 2 h, NR	PNS (*n* = 10, ip)		PNS 100 mg/kg		30 min before and 6 h after surgery		1. NDS (ZL, 24 h) 2. TGF-β1 in the ischemic region by immunohistochemistry and RT-PCR	
Wang Ping Wang et al. (2018b)	2018		SD rats, 240–280 g, M		MCAO (ZL), NR, NR	1. Edaravone (*n* = 7, ip) 2. PNS-R (*n* = 11, ip) 3. PNS-before R (*n* = 7, ip)		Edaravone 5 mg/kg PNS 10 mg/kg PNS 10 mg/kg		At the beginning of reperfusion At the beginning of reperfusion 10 min before reperfusion		1. NDS (ZL, 24 h) 2. CIV (TTC, 24 h)	
Zhang Yongquan Zhang et al. (2008)	2008		SPF SD rats,260–300 g, M		MCAO (ZL), 2 h, NR	1. PNS-L (subgroup *n* = 18, ip) 2. PNS-H (subgroup *n* = 18, ip) 3. Ligustrazine (subgroup *n* = 18, ig)		PNS 25 mg/kg PNS 50 mg/kg Ligustrazine 80 mg/kg		30 min before and 6 h after surgery		1. NDS (ZL, 24 h) 2. CIV (TTC, 24 h) 3. BDNF, neuron apoptosis	

AST IV, astragalosides IV; Bax, Bcl⁃2 associated x protein; BBB, blood-brain barrier; Bcl-2, B⁃cell lymphoma⁃2; BDNF, brain-derived neurotropic factor; Caspase-3, cysteine aspartate protease; CIV, cerebral infarct volume; F, female; GAP-43, growth associated protein-43; GFAP, glial fibrillary acidic protein; H, high dosage; ip, intraperitoneal injection; ig, intragastric administration; IL-1β, interleukin-lβ; IL-6, interleukin-6; IL-8, interleukin-8; IL-10, interleukin⁃10; iv, intravenous administration; L, low dosage; LN, laminin; M, male; M, medium dosage; MAP-2, microtubule-associated protein 2; MCAO, middle cerebral artery occlusion; NDS, neurologic deficit scores; NeuN, neuronal nuclei; NR, no report; NSE, neuron specific enolase; NVU, neurovascular unit; PNS, *Panax notoginseng* (Burkill) F.H. Chen saponins; PNFS, *Panax notoginseng* (Burkill) F.H. Chen flower saponins; PNLS, *Panax notoginseng* (Burkill) F.H. Chen leaf saponins; PSD, postsynaptic density; SS combination, saponins of *Panax notoginseng* (Burkill) F.H. Chen leaves and *Crataegus Pinnatifida* Bge. leaves; Syp, synaptophysin; TGF-β1, transforming growth factor; TNF-α, tumor necrosis factor-α; TTC, 2,3,5-triphenyltetrazolium chloride; VEGF, Vascular endothelial growth factor.

**TABLE 4:** A summary table of the preparations used in the selected literatures.

Study	Year	Extract/preparation	Source	Compound, concentration	Quality control reported? (Y/N)	Chemical analysis reported? (Y/N)
Chen Jie	2015	PNS	Commercial supplier	PNS, NR	Y-Prepared according to CP	Y-HPLC
Chen Ming	2007	Xuesaitong injection (lyophilized)	Commercial supplier	PNS, NR	Y-Prepared according to DSC	Y-HPLC
He Yan	2017	1. Xuesaitong capsule/tablet 2. PNLS	1. Commercial Supplier 2. Purified by Academy Of Chinese Medicine Sciences of Jilin Province	PNLS, NR	1. Y-Prepared according to DSC 2. N	1. Y-HPLC 2. N
Jiang Huihui	2015	PNS	Commercial supplier	PNS, NR	Y-Prepared according to CP	Y-HPLC
Li Jingxian	2017	PNS	Commercial supplier	PNS, NR	Y-Prepared according to CP	Y-HPLC
Lin Jun	2019	Xuesaitong injection (lyophilized)	Commercial supplier	PNS, 100/150/250 mg	Y-Prepared according to DSC	Y-HPLC
Liu Lixing	2014	Xuesaitong injection (lyophilized)	Commercial supplier	PNS, 400 mg	Y-Prepared according to DSC	Y-HPLC
Liu Yinhua	2018	PNFS	Commercial supplier	PNFS, NR	N	N
Meng Lanqing	2014	Xuesaitong injection	Commercial supplier	PNS, NR	Y-Prepared according to DSC	Y-HPLC
Sun Xiaoqing	2010	PNS	Commercial supplier	PNS, NR	Y-Prepared according to CP	Y-HPLC
Tang Jingshu	2011	Xuesaitong injection	Commercial supplier	PNS, NR	Y-Prepared according to DSC	Y-HPLC
Wang Jiehua	2014	PNS	Commercial supplier	PNS, NR	Y-Prepared according to CP	Y-HPLC
Wang Ping	2018	PNS	Commercial supplier	PNS, NR	Y-Prepared according to CP	Y-HPLC
Zhang Yongquan	2008	Xuesaitong injection (lyophilized)	Commercial supplier	PNS, NR	Y-Prepared according to DSC	Y-HPLC

CP, Chinese Pharmacopoeia; DSC, Drug Standards of China; NR, no report; PNS, Panax notoginseng (Burkill) F.H. Chen saponins; PNFS, Panax notoginseng (Burkill) F.H. Chen flower saponins; PNLS, Panax notoginseng (Burkill) F. H. Chen leaf saponins.

### The Methodological Quality of the Included Studies

**FIGURE 3:**
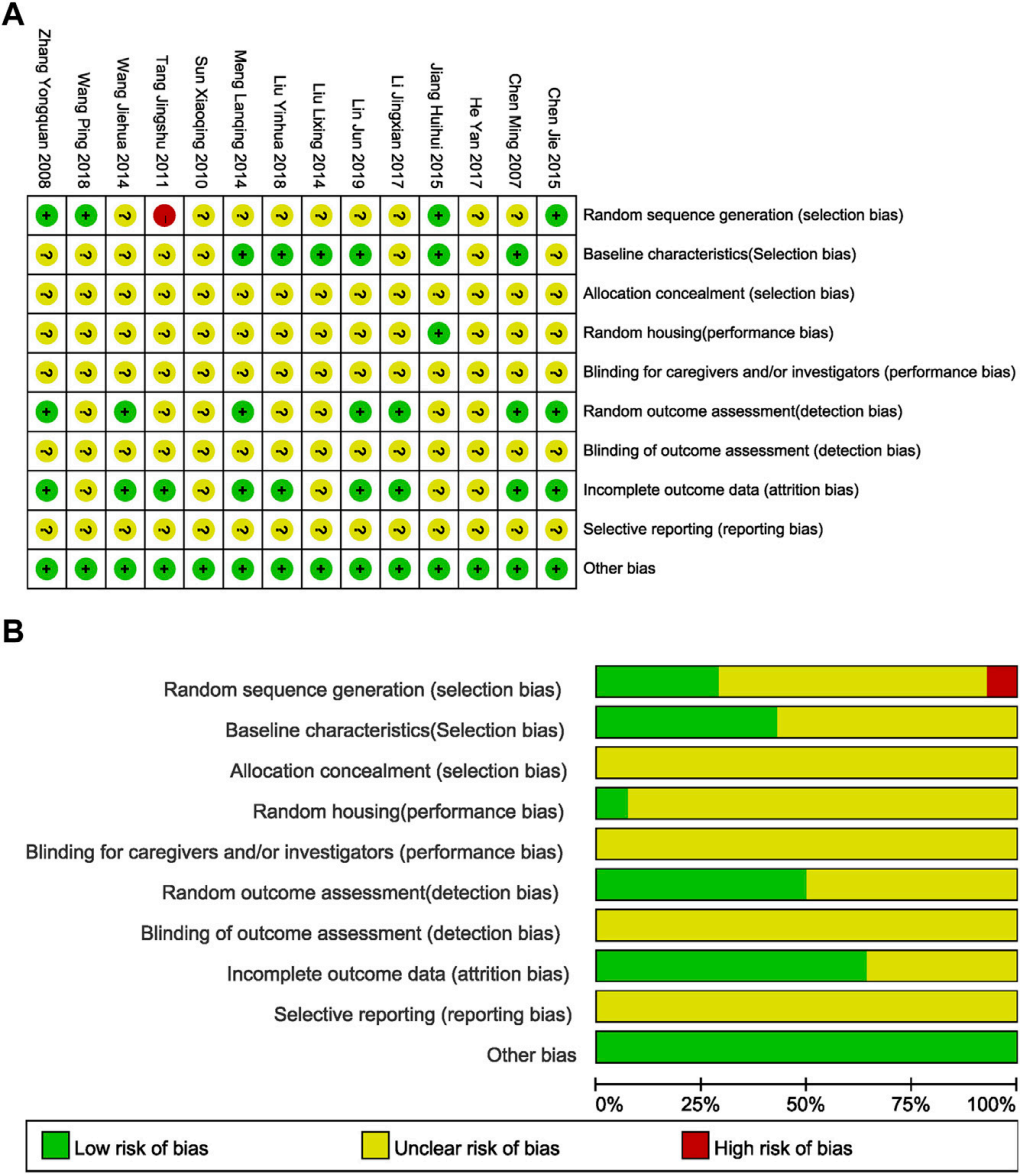
Evaluation of literature quality results obtained through SYRCLE’s Risk of Bias based on the Cochrane tool. **(A)** Risk of bias summary: review authors’ judgements about each risk of bias item for each included study. **(B)** Risk of bias graph: review authors' judgements about each risk of bias item presented as percentages across all included studies.

With the SYRCLE’s Risk of Bias tool for research and analysis, 4/14 (28.57%) reports illustrated the randomization method, 1 (7.14%) of the reports did not express the randomization method, and the other reports only described “randomization” without specifying the randomization method, which was judged as “unclear risk of bias.” For the rat models with I/R, six articles (42.86%) clearly expressed that rats could be included in the experiment only after being evaluated and qualified in order to ensure uniform experimental baseline standards. Other articles did not clearly explain this requirement (for example, no statement was made for the randomness of the surgical process in the group undergoing surgery). In the field of random housing, only one document detailed the consistency and randomness of the environment in which experimental rats were housed. Seven articles (50.00%) tested all the test rats in all groups for the target index of this meta-analysis, while the other documents were not completely tested based on the initial grouping and did not explain the randomness of the test results. Nine articles (64.29%) described the absence of lost follow-up data. None of the studies expressed any other deviation, so they were all evaluated as “low risk.” Additionally, none of the literatures showed whether allocation was concealed and whether they were blinded for caregivers and investigators, blinded outcome assessment or selective reporting (Figure 3). The published literatures also have limitations in the design and implementation of animal experiment methodology and need to be ameliorated to ensure the feasibility of transforming basic research into clinical study.

With the modified CAMARADES for quality evaluation, the included studies scored an average of 2.36 (1–4 points) out of 10 points, indicating that the animal trials remain evolving to improve consistency and guidance. For cerebral I/R rat testing, only three articles considered the monitoring of physiological parameters during surgery, three articles examined the influence of doses of cerebral ischemia effect indicators, and two articles calculated the necessary sample size to achieve the specified quality of the test. No studies clearly stated the blinded assessment of behavioral outcome, potential conflict of interests, compliance with animal welfare regulations, and the use of a suitable animal model (Table 5).

**TABLE 5:** CAMARADES evaluation results.

	(A)	(B)	(C)	(D)	(E)	(F)	(G)	(H)	(I)	(J)	Total
Chen Jie	✔	✔			✔			✔			4
Chen Ming	✔										1
He Yan	✔							✔			2
Jiang Huihui	✔	✔	✔					✔			4
Li Jingxian	✔							✔			2
Lin Jun	✔										1
Liu Lixing	✔		✔					✔			3
Liu Yinhua	✔		✔			✔					3
Meng Lanqing	✔					✔		✔			3
Sun Xiaoqing	✔										1
Tang Jingshu	✔				✔			✔			3
Wang Jiehua	✔										1
Wang Ping	✔	✔			✔						3
Zhang Yongquan	✔	✔									2

**(A)** Peer-reviewed publications; **(B)** randomization of subjects into treatment groups; **(C)** assessment of dose-response relationship; **(D)** blinded assessment of behavioral outcome; **(E)** monitoring of physiological parameters such as body temperature; **(F)** calculation of necessary sample size to achieve sufficient power; **(G)** statement of compliance with animal welfare regulations; **(H)** avoidance of anesthetic agents with marked intrinsic neuroprotective properties; **(I)** statement of potential conflict of interests; **(J)** use of a suitable animal model.

### Overall Efficacy of PNS on NDS

**FIGURE 4:**
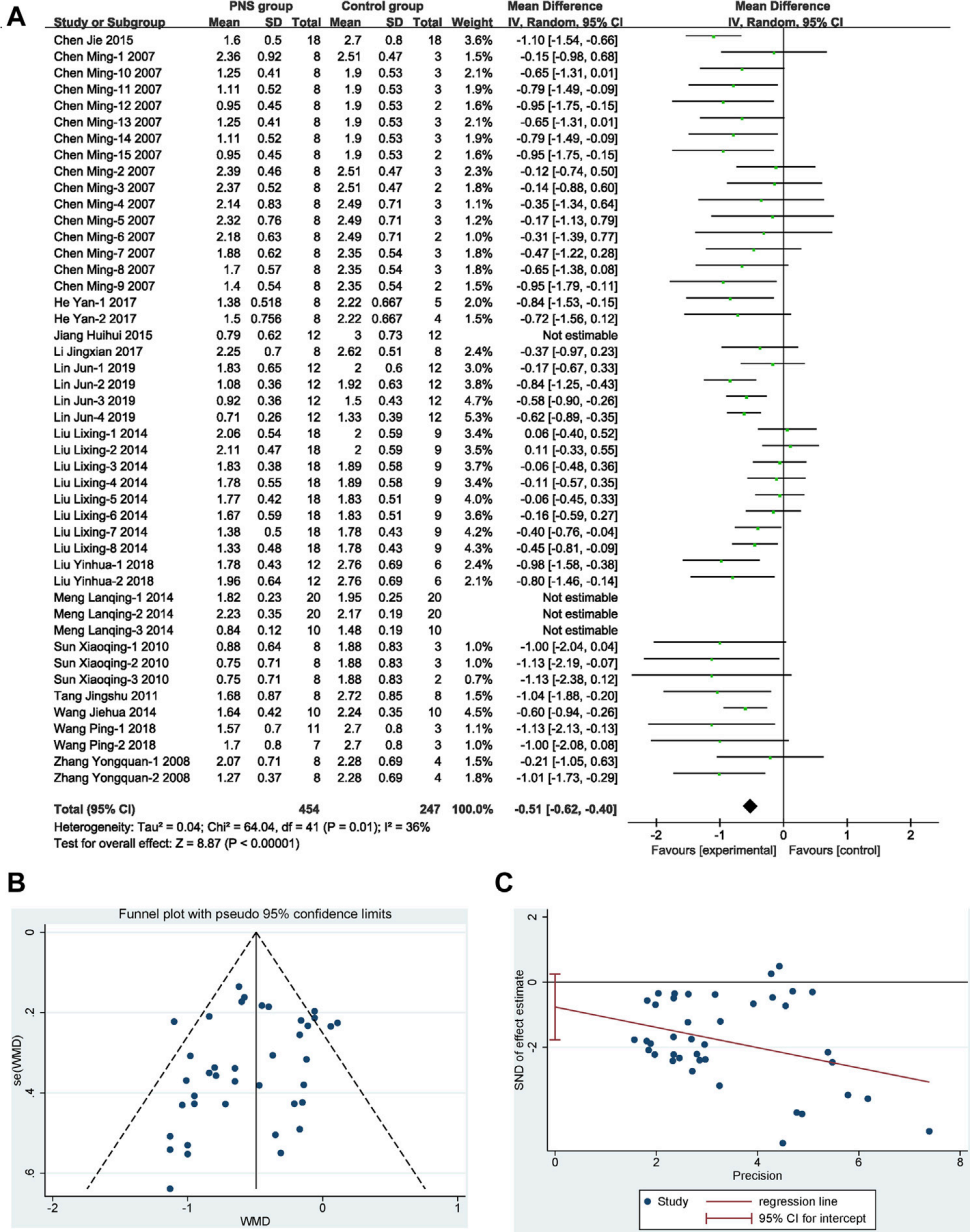
Overall efficacy of PNS on NDS. **(A)** Results of the meta-analysis visualized in a forest plot. Forest plot adopted mean difference and its corresponding 95% CI based on the random-effects model (Inverse Variance). It is showed that PNS significantly reduced the score of NDS with mild heterogeneity by removing the outlier studies. **(B)** Assessing of publication bias in a funnel plot. No publication bias could be detected from the symmetrical funnel plot. **(C)** Bias assessment plot by Egger’s test. Egger’s weighted regression suggested no publication bias for all analyses.

Forty-six (46) study groups were included in this analysis. There was moderate heterogeneity (I^2^ = 72%, *p* < 0.1). A sensitivity analysis of the included literature revealed that two documents (Meng et al., 2014; Jiang et al., 2015) had a greater impact on heterogeneity, so two outlier documents were removed by reevaluation. Finally, 42 study groups with 701 participants reported data on NDS, and the treatment with PNS reduced the score of NDS by 12.75% compared with the control group. The difference was statistically significant (MD −0.51, 95% CI: −0.62 to −0.40; *p* < 0.01) with heterogeneity (I^2^ = 36%, *p* = 0.01) (Figure 4A). The symmetrical funnel plot (Figure 4B) showed no published bias, and Egger’s regression test (Figure 4C) for symmetry was not significant (*p* = 0.098).

### Prespecified Subgroup Analysis

In the subgroup factors analysis, the NDS was used as a surrogate marker for efficacy. The factors of ischemic duration, drug dosage, and intervention time had significant impacts on NDS (subgroup heterogeneity values were I^2^ = 74.8, 78.8, and 78.1%, respectively) (Table 6), while the administration duration had nonsignificant impact (subgroup heterogeneity I^2^ = 38.4%).

**TABLE 6:** Subgroup analysis of PNS according to NDS.

Group or subgroup	PNS group (*n*)	Control group (*n*)	Mean difference (MD)	Difference in NDS (%)	Overall effect test	Heterogeneity
Ischemia time						
Ischemia 1 h*	40	17	−0.91 [−1.32, −0.50]	22.75	Z = 4.37 (*p* < 0.0001)	Tau^2^ = 0.00; Chi^2^ = 0.55, df = 4 (*p* = 0.97); I^2^ = 0%
Ischemia 2 h	180	92	−0.63 [−0.78, −0.49]	15.75	Z = 8.54 (*p* < 0.00001)	Tau^2^ = 0.00; Chi^2^ = 17.62, df =20 (*p* = 0.61); I^2^ = 0%
Ischemia unknown	234	138	−0.38 [−0.55, −0.21]	9.50	Z = 4.44 (*p* < 0.00001)	Tau^2^=0.06; Chi^2^=34.59, df=15 (*p* = 0.003); I^2^ = 57%
Subgroup differences						Chi^2^=7.92, df = 2 (*p* = 0.02); I^2^ = 74.8%
Dosage						
D_1_ < 25 mg/kg*	26	10	−0.92 [−1.47, −0.37]	23.00	Z = 3.26 (*p* = 0.001)	Tau^2^ = 0.00; Chi^2^ = 0.41, df=2 (*p* = 0.81); I^2^ = 0%
25 mg/kg ≤ D_2_ < 50 mg/kg	96	48	−0.28 [−0.53, −0.04]	7.00	Z = 2.24 (*p* = 0.03)	Tau^2^ = 0.04; Chi^2^ = 9.10, df = 6 (*p* = 0.17); I^2^ = 34%
50 mg/kg ≤ D_3_ < 100 mg/kg	236	135	−0.45 [−0.59, −0.32]	11.25	Z = 6.58 (*p* < 0.00001)	Tau^2^ = 0.03; Chi^2^ = 28.76, df = 21 (*p* = 0.12); I^2^ = 27%
100 mg/kg ≤ D_4_ < 150 mg/kg	96	54	−0.80 [−0.99, −0.60]	20.00	Z = 7.98 (*p* < 0.00001)	Tau^2^ = 0.00; Chi^2^ = 8.29, df = 9 (*p* = 0.51); I^2^ = 0%
Subgroup differences						Chi^2^ = 14.17, df = 3 (*p* = 0.003); I^2^ = 78.8%
Intervention time						
Before occlusion	72	35	−0.60 [−0.84, −0.35]	15.00	Z = 4.77 (*p* < 0.00001)	Tau^2^ = 0.00; Chi^2^ = 3.97, df = 7 (*p* = 0.78); I^2^ = 0%
After occlusion	274	149	−0.40 [−0.55, −0.25]	10.00	Z = 5.23 (*p* < 0.00001)	Tau^2^ = 0.05; Chi^2^ = 37.90, df = 21 (*p* = 0.01); I^2^ = 45%
Before and after occlusion*	108	63	−0.76 [−0.94, −0.57]	19.00	Z = 8.08 (*p* < 0.00001)	Tau^2^ = 0.00; Chi^2^ = 9.77, df = 11 (*p* = 0.55); I^2^ = 0%
Subgroup differences						Chi^2^ = 9.14, df = 2 (*p* = 0.01); I^2^ = 78.1%
Duration						
Duration T = 1–3 days	390	202	−0.47 [−0.60, −0.34]	11.75	Z = 7.10 (*p* < 0.00001)	Tau^2^ = 0.05; Chi^2^ = 57.07, df = 35 (*p* = 0.01); I^2^ = 39%
Duration T = 4–7 days	28	21	−0.64 [−0.91, −0.36]	16.00	Z = 4.58 (*p* < 0.00001)	Tau^2^ = 0.00; Chi^2^ = 0.50, df = 2 (*p* = 0.78); I^2^ = 0%
Duration T > 7 days*	36	24	−0.69 [−0.92, −0.46]	17.25	Z = 5.96 (*p* < 0.00001)	Tau^2^ = 0.00; Chi^2^ = 1.26, df = 2 (*p* = 0.53); I^2^ = 0%
Subgroup differences						Chi^2^ = 3.24, df = 2 (*p* = 0.20); I^2^ = 38.4%
Total	454	247	−0.51 [−0.62, −0.40]	12.75	Z = 8.87 (*p* < 0.00001)	Tau^2^ = 0.04; Chi^2^ = 64.04, df = 41(*p* = 0.01); I^2^ = 36%

A random-effects model was used to calculate the pooled estimates of the mean differences and explore the impact of ischemia duration, dosage, intervention time, and duration of administration. The ischemia time 1 h, D1 < 25 mg/kg, before and after occlusion and duration T > 7 days groups were the most effective groups within each exploratory group and were marked with *.

Sorts of ischemic durations showed different treatment effects. In the rat experiments, restoration of blood perfusion and intervention within 1 h (22.75%) and 2 h (15.75%) improved the NDS compared with the average value (12.75%). This finding also suggested that the shorter the ischemic time is, the more prominent the treatment advantage would be in the later treatment sessions.

Drug dosage is one of the key factors that reflect the different efficacy of PNS. When the dose given to rats was D_1_ (23.0%) or D_4_ (20.0%), its therapeutic effect on NDS was better than that in the D_2_/D_3_ dosage groups (7 and 11.25%). That is to say, the therapeutic effect lower than 25 or 100–150 mg/kg was better than 25–100 mg/kg. It is shown that the association between the PNS dosage and NDS is materialized by a characteristic inverted U-shaped dose-response curve, revealing that the MCAO models with low- and high-dosage PNS both had better efficacy of NDS.

The time of PNS intervention is one of the factors affecting the late recovery of MCAO. The before occlusion intervention group (15.0%) and the before and after occlusion intervention group (19.0%) had higher NDS-improving efficacy than the after occlusion intervention group (10.0%), and the before and after occlusion intervention group was even more efficacious. The results suggested that preventive and comprehensive PNS administration had better therapeutic effects on the neurobehavioral effects caused by cerebral ischemic injury compared with the therapeutic administration. Additionally, there was moderate heterogeneity in the treatment administration group and the comprehensive treatment group. The reason may be that they were inconsistent in postoperative observation time, and the recovery status fluctuated greatly, while the preventive administration group observed time concentration within 24 h.

The administration duration is also related to the prognosis of MCAO and the level of NDS. The effect of NDS in the subgroups given drugs for more than 4 days (16.0 and 17.25%) was better than that of the subgroups given drugs for 1–3 days (11.75%), and the heterogeneity among subgroups was low. It is suggested that clinical medication could be taken for a long time according to the patient conditions.

### Possible Protection Efficacy and Mechanism Analysis

**FIGURE 5:**
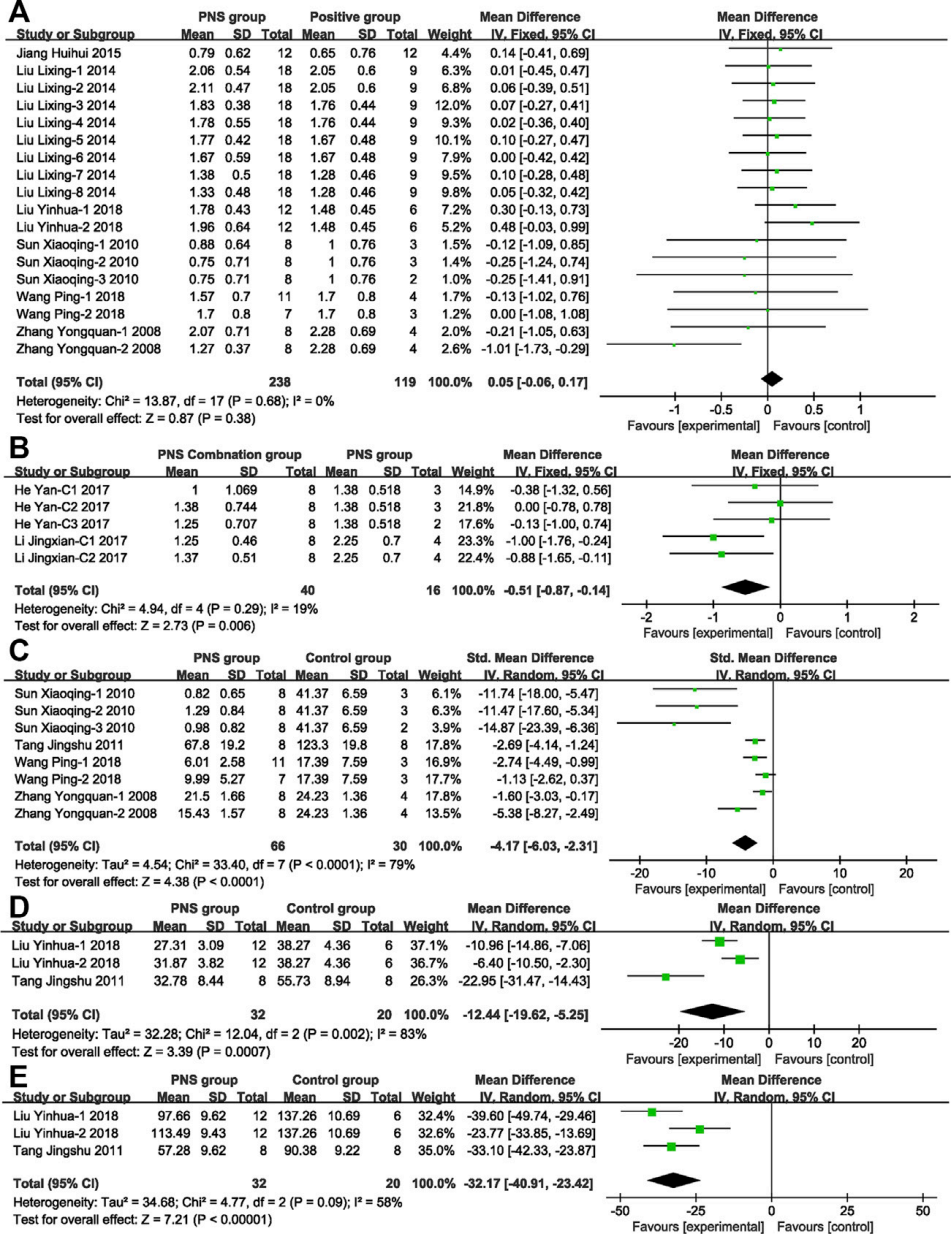
Forest plots of possible protection efficacy and mechanism analysis. **(A)** PNS vs. positive drug by using fixed-effect model (MD) analysis on NDS exhibits no statistical significance or heterogeneity. **(B)** PNS concomitant medication vs. PNS by using fixed-effect model analysis (MD) on NDS exhibits statistical significance and no heterogeneity. **(C)** PNS vs. control group by using random-effect model analysis (SMD) on CIV exhibits statistical significance and high heterogeneity. **(D)** PNS vs. control group by using random-effect model analysis (MD) on IL-1β exhibits statistical significance and high heterogeneity. **(E)** PNS vs. control group by using random-effect model analysis (MD) on TNF-α exhibits statistical significance and moderate heterogeneity.

Figure 5A compares the NDS between PNS group and positive control drug group. By comparing the effects of PNS and positive control drugs on the NDS of MCAO rats, nine studies with 357 rats (PNS group: *n* = 238, positive drug group: *n* = 119) were included. PNS had no statistical significance compared with the positive control drugs, and there was no heterogeneity in the study, suggesting that the PNS has a better effect on I/R than positive control drugs.

Figure 5B compares the NDS score in PNS combination group and PNS group. By comparing the efficacy of PNS combination treatment (combined with *Crataegus Pinnatifida* Bge. leaf extract or astragalosides IV) and PNS alone treatment, five studies with 56 participants were included (PNS combination group: *n* = 40, PNS group *n* = 16). The PNS combination group was 12.75% better than the PNS alone group, indicating that the clinical application of PNS combinations, according to the patient conditions, can effectively improve adverse symptoms.

Figure 5C compares the CIV of the PNS group and positive control drug group. Nine articles with 132 participants showed that PNS could significantly reduce the area of cerebral ischemic infarction by 4.17 and alleviate the pathological symptoms of the ischemic model. Various studies have analyzed the mechanism of PNS to protect nerves.

The mechanisms of the drugs were analyzed. A meta-analysis of the inflammatory factors IL-1β (Figure 5D) and TNF-α (Figure 5E) in the included literature was performed. The study showed that PNS significantly inhibited the release of the inflammatory factors up to 12.44 pg ml^−1^ (IL-1β) and 32.17 pg ml^−1^ (TNF-α).

## Discussion

We analyzed the focal MCAO rat model (Longa et al., 1989), which is relatively noninvasive with reversible regional cerebral ischemia. The model simulated the clinical pathogenesis and treatment of focal cerebral I/R patients and showed good evaluation effect. To reduce bias and ensure the validity of the data entered, we selected the unilateral MCAO model instead of the bilateral vertebral artery occlusion model, which also developed regional infarction accompanied by a series of pathological changes. The clinical evaluation of stroke includes a neurological examination, monitoring of vital signs, blood analysis, immediate neurovascular (vascular and brain) imaging, and cardiovascular investigations (Boulanger et al., 2018). All suspected patients with acute stroke should be assessed for neurological impairments and functional limitations (cognitive evaluation, screening for depression, and assistance with activities of daily living) and undergo brain imaging with NCCT or MRI (Boulanger et al., 2018). Therefore, we determine the efficacy of PNS with NDS and CIV, which was indicated to be significantly effective in the meta-analysis.

Statistical experiments (Perel et al., 2007; Blocker et al., 2019) showed that there was a significant difference between animal study results and clinical treatment effects in the treatment of stroke. Indeed, it objectively presents the probability of inconsistency between preclinical study and clinical disease treatment results. Although we cannot accurately project the value of the results of preclinical experiments, animal experiments are needed to further examine the biological mechanisms (Pound and Ritskes-Hoitinga, 2020). On the other hand, the lack of consistency between preclinical experiments and clinical trials may be due to random errors, bias, or the differentiation of animal models and human diseases. Consequently, in order to bridge the preclinical and clinical study, we need to further improve the quality of animal study methods, explore more systematic analysis of experimental data, and strengthen joint study between clinical and animal investigators (Bahadoran et al., 2020).

Based on the above analysis, the PNS drug dosage and NDS are characterized by an inverted U-shaped effect curve, which not only provides evidences for clinical applications, but also provides ideas for in-depth research of drug mechanisms. The study by Gold et al. (Gold and Van Buskirk, 1976) showed that the administration of adrenocorticotrophic hormone has an inverted-U dose-response curve characteristic on the memory enhancement of animals. Knauber et al. (Knauber and Muller, 2000) found that when prazosin was subchronically given to the elder rats to evaluate the learning capabilities and memory retention, the drug dose and the results of passive avoidance learning showed the characteristics of U-shaped dose-response curve by upregulating the α1-receptor density. Regarding the characteristics of drug dosage, it may be the potential way to further explore the mechanism of PNS and cerebral ischemia. Concomitantly, the dosage analysis of the animal experiments shows that human doses below 4.03 or 16.13–24.19 mg/kg can exert significant influence, but they are inconsistent with the current clinical utility and cause individual adverse effects at high doses. According to the 2008 clinical analysis of PNS in the Cochrane Database of Systematic Reviews, PNS can significantly reduce mortality and drug dependence in the clinic, reduce patient symptoms, and improve behavior scores. PNS are mainly used as an injection, and the drug dosage range is nearly 6.67–13.33 mg/kg over 14–28 days (Jiliang et al., 2005; Xi-hong and Min-jiu, 2009; Jin and Zheng, 2011; Gui et al., 2013; Li et al., 2016). This dose is not included in the optimal drug range from the experimental animal analysis. In Liu HL’s study (Jiliang et al., 2005), the drug was used at a dose of 400–800 mg/day, and three cases (a total of 120 cases in the group) had allergic dermatitis. In addition, the adverse reactions of PNS injections (Wan, 2013; Huang, 2018) from 1998 to 2017 showed that the damage of PNS injections involved multiple systems in the body, mainly damage to the skin and digestive, respiratory, and nervous systems and anaphylactic shock in severe cases. No effect of the dose was stated. Therefore, it is necessary to further explore the optimal dosage of PNS in the clinic, strengthen the monitoring of adverse reactions, and reduce the risk of medication for patients.

The study proves that PNS interventions at various stages of the disease can achieve significant results, covering primordial prevention (before occlusion), primary prevention (before and after occlusion), and secondary prevention (after occlusion). Cheng (Cheng et al., 2015) points out that intervention in the early diagnosis of lacunar infarction (within 24 h) increases the relative cerebral blood flow at discharge and improves neurological deficits in elderly patients. Zhang (Zhang et al., 2010) shows that the recurrence rate of ischemic stroke in the PNS group (5.83%) is significantly lower than that in the control group (17.82%).

The rat experiments together show that the effects are more prominent as the PNS treatment prolongs. Significant effects were achieved in rat models after treatment for more than four consecutive days, and the effect of more than 7 days improved even more. Clinically, the PNS medication cycle varies from 7 to 21 days, and all durations showed the protective effect on brain nerve cells (Wu et al., 2002; Feng et al., 2011; Gao et al., 2015). Dong XL (Dong et al., 2005) compared the drug usage of PNS for 7 and 21 days and measured blood specific viscosity (BSV), plasma fibrinogen, erythrocyte rigidity, and K value of erythrocyte sedimentation rate equation, extrapolating to the cumulative effects of treatments over time.

This study indicates that PNS has excellent therapeutic effects that are analogous to those of positive control drugs, but its mechanism is worthy of extensive study and verification. Our study shows that PNS can alleviate MCAO symptoms by reducing the inflammatory response. However, AIS causes neuronal cell death through complex pathological changes, including the excessive influx of Ca^2+^, the formation of reactive oxygen species (ROS), and the dysfunction of mitochondrial. Edaravone captures and reduces excessive ROS to prevent brain damage (Matsumoto et al., 2018). Nimodipine is a calcium channel blocker that can significantly reduce the protein levels of caspase-3 and Bax in rat brain tissue and may inhibit the release of Ca^2+^ (Yang et al., 2006). The therapeutic mechanism of PNS (Wang et al., 2016; Zhao et al., 2016; Wang et al., 2018a) was based on the decrease in the levels of ROS and malondialdehyde (MDA); the increase in the activity of glutathione peroxidase (GSH-Px), catalase (CAT), and superoxide dismutase (SOD); and the increase in cell viability and mitochondrial membrane potential. Additionally, PNS (Li et al., 2009) significantly attenuated the expression of caspase-3 and caspase-1 compared with the model group to achieve the neuroprotective effect on focal ischemia. There is increasing evidence that PNS treats MCAO by protecting nerve cells through multiple pathways and multiple targets.

In the present study, the favorable evidence of the concomitant medication also strengthens our confidence in treating MCAO with PNS combined with other traditional Chinese medicine ingredients (combined with *Crataegus Pinnatifida* Bge. leaf extract or astragalosides IV). By synthetically analyzing the animal and clinical trials, it is found that the concomitant medication of PNS can indeed synergistically enhance the efficacy and has favorable safety. Feng CL (Feng et al., 2011) has confirmed that AST IV combined with PNS improves cerebral vascular microcirculation, increases cerebral vascular blood flow, enhances the ability of brain cells to resist hypoxia, etc. AST IV combined with PNS has a protective effect on ischemic brain cells. Xue (Xue, 2018) has shown that edaravone combined with PNS significantly reduces the score of National Institutes of Health Stroke Scale (NIHSS). Hyperbaric oxygenation (HBO) (Wu et al., 2002; Dong et al., 2005) associated with PNS has rapid effects with few adverse reactions in the treatment of abnormal hemorheology in patients with ischemia cerebral vessel disease. The PNS administration plus intravenous thrombolysis with recombinant tissue-type plasminogen activator (rt-PA) (Li et al., 2016) can reduce I/R injury in patients with AIS by reducing the occurrence of hemorrhagic transformation and improving the prognosis with good safety. Zhang (Zhang et al., 2010) has showed that the combination of PNS and aspirin is better than aspirin alone in preventing recurrence of ischemic stroke.

In addition to being an effective adjuvant therapy in clinical ischemic stroke patients, PNS exhibits broad biological activities (Zhang, 2004; Zhang et al., 2019) and has obvious effects on cardiovascular system diseases, the endocrine system, and improved nerve function. Spontaneous intracerebral hemorrhage (ICH) patients (Luo et al., 2010a; Luo et al., 2010b; Luo et al., 2018) who were treated with Xueshuantong injection (175 mg/day) for two weeks exhibited increased hematoma absorption, improved recovery of neurological function, and reduced inflammatory response. Song et al. (2013) showed that photodynamic therapy (PDT) combined with PNS in the treatment of age-related macular degeneration (AMD) and choroidal neovascularization (CNV) has a reliable effect and can be used in clinical application. PNS (Deng, 2010) can significantly improve ischemia in diabetic foot patients by reducing the amount of angiotensin, reducing purulent secretions, and promoting healing of the ulcer area. Therefore, PNS can play a comprehensive protective role in patient management.

## Limitations

In this study analysis, all the studies included the rat models treated with PNS for ischemic stroke. The rats have relatively low cost, good physiological responses, and available genetic techniques. However, there have been failure cases of translation from preclinical studies to clinical trials reported (Perel et al., 2007; Hossmann, 2009). In addition, no animal models of complications were selected in this systematic research literature, and this differed from the actual clinical situation. Therefore, other animal models need to be considered; investigators should choose the models that are more comparable to the development of human physiology and pathology, that are related to the pathogenesis of ischemic stroke, that have a more accurate experimental design of treatment window duration, and that have a sufficient basis for the translation of preclinical findings (Kaiser and West, 2020).

The included literature has problems with methodological irregularities and incomplete study reports, which may affect the validity of the results and conclusions. The common limitations of these studies are lack in describing participant randomization, allocation, results evaluation by the evaluators, limited available data on the main results, and unclear methods and dosage of traditional Chinese formulation. Therefore, the included reports are rated as high-risk or unclear-risk, which requires investigators to focus on improving their methodology and report quality in animal experiments to provide high-quality evidence.

## Conclusion

A total of 14 articles were included in this meta-analysis. After heterogeneity judgment and sensitivity analysis, 43 study groups with 749 participants were finally included in our study. More reliable preclinical evidence was obtained based on the analysis of the NDS, CIV, and inflammatory factors TNF-α and IL-1β.

The comprehensive analysis of PNS intervention in MCAO rat models shows that early application of PNS intervention at all stages of the disease has significant effects on disease treatment, including primordial prevention, primary prevention, and secondary prevention. If the dosage of administration in rats is controlled within lower than 25 or 100–150 mg/kg and the drug is administered for more than 4 days, rat behavioral scores can be significantly changed, brain tissue can be protected from pathological changes, and the content of inflammatory factors in the body can be reduced. The effect of combined PNS and astragalosides IV or *Crataegus pinnatifida* Bge. leaf extract was more significant than that of PNS alone. It is worthwhile to transform PNS from laboratory to clinic.

Most of the studies included could not be fully assessed since there is an unclear risk of bias for the vast majority of the parameters. Consequently, the overarching outcome of this meta-analysis remains inconclusive and we urgently need much better designed studies.
